# Comparison of allogeneic hematopoietic stem cell transplantation and TKI combined with chemotherapy for adult philadelphia chromosome positive acute lymphoblastic leukemia: a systematic review and meta‐analysis

**DOI:** 10.1002/cam4.4413

**Published:** 2021-11-11

**Authors:** Qiang Zeng, Bing Xiang, Zhigang Liu

**Affiliations:** ^1^ Department of Hematology West China Hospital Sichuan University Chengdu China

**Keywords:** allogeneic hematopoietic stem cell transplantation, meta‐analysis, Philadelphia chromosome positive acute lymphoblastic leukemia, tyrosine kinase inhibitor

## Abstract

**Objective:**

This study seeks to clarify whether allogeneic hematopoietic stem cell transplantation (allo‐HSCT) is necessary for adult patients with Philadelphia chromosome‐positive acute lymphoblastic leukemia (Ph+ ALL) in post‐remission based on a comparison with tyrosine kinase inhibitor (TKI) combined with chemotherapy.

**Methods:**

We searched the Pubmed, Embase, and Web of Science databases and limited the date range for the studies from January 2010 to August 2020. A hazard ratio (HR) with a 95% confidence interval (CI) was employed to assess overall survival (OS) and relapse‐free survival (RFS), and an odds ratio (OR) with a 95% CI was used to evaluate the ratio of non‐relapsed mortality (NRM) and non‐relapsed survival (NRS). All analyses were conducted with Stata software 16.0 and Revman 5.3.

**Results:**

Fifteen studies, totaling 959 patients, were included in our analysis. Among those patients, 473 underwent allo‐HSCT, and 486 received TKI plus chemotherapy. The pooled results showed no difference in OS between outcomes for patients receiving TKI plus chemotherapy and those treated with allo‐HSCT (HR = 0.76, 95% CI [0.51–1.12], *p *= 0.16). Patients undergoing allo‐HSCT did better than those receiving TKI plus chemotherapy regarding RFS (HR = 0.48, 95% CI [0.37–0.63], *p* = 0.00), and NRS (OR = 2.64, 95% CI [1.25–5.57], *p *= 0.00). The NRM rate of the TKI plus chemotherapy group was significantly lower than the allo‐HSCT group (OR = 2.33, 95% CI [1.51–3.59], *p *= 0.00).

**Conclusion:**

TKI combined with chemotherapy can be considered a post‐remission treatment option for adult Ph+ ALL patients who are ineligible for allo‐HSCT. However, more prospective studies with large sample sizes should be carried out in the future.

## Graphical abstract

NRM occurred more frequently in the allo‐HSCT patients than in those undergoing TKI plus chemotherapy. TKI combined with chemotherapy can be considered a post‐remission treatment option for adult Ph+ ALL patients who are ineligible for allo‐HSCT.

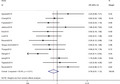

## 1 INTRODUCTION

Philadelphia chromosome‐positive acute lymphoblastic leukemia (Ph+ ALL) is a high‐risk lymphocyte tumor characterized by the presence of t(9,22), which contributes to a poor outcome.^1^, ^2^ The incidence rate of Ph+ ALL increases with age.^3^, ^4^ Among adult ALL patients, 20–30% are identified with t(9,22) when diagnosed.^5^ In the pre‐tyrosine kinase inhibitor (TKI) era, high‐intensive chemotherapy followed by allogeneic hematopoietic stem cell transplantation (allo‐HSCT) had been the standard treatment regimen for Ph+ ALL patients.^6^ However, many patients only receive continuous high‐intensity chemotherapy instead of undergoing allo‐HSCT due to the lack of available donors, advanced age, and economic issues. Among these patients, the long‐term survival rate is only 10%, and most deaths are due to recurrence and complications caused by long‐term intensive chemotherapy.^7^ Allo‐HSCT indeed prolongs life survival by comparison. Nevertheless, since post‐transplantation patients are at risk of acute or chronic graft‐versus‐host disease (GvHD), various infections, and relapse, the overall survival (OS) of adult patients with Ph+ ALL remains around 50% or lower.^8^, ^9^, ^10^

Fortunately, patient prognosis has improved since TKI was first introduced. With TKI added to induction chemotherapy, the hematological complete remission (HCR) rate has surpassed 90% in Ph+ ALL patients,^11^ which helps more patients an opportunity to receive allo‐HSCT. Additionally, patients live longer and do not relapse after TKI is applied to consolidation and maintenance therapy or post‐transplantation treatment.^12^, ^13^, ^14^, ^15^, ^16^ Newer generation TKI can help patients with Ph+ ALL achieve deeper remission as well.^17^, ^18^, ^19^, ^20^, ^21^, ^22^ Numerous studies demonstrated that TKI was the key to maintaining long‐term complete remission status. Historically, chronic myeloid leukemia (CML) has been a disease with a poor prognosis, and allo‐HSCT has been the only potentially curative option.^23^, ^24^ With the advent of TKI, however, CML has become a curable disease without transplantation.^25^, ^26^, ^27^ As the integration of TKI greatly improves the prognosis of Ph+ ALL patients, is allo‐HSCT clinically imperative among patients with Ph+ ALL? Most studies reported the promising conclusion that TKI combined with chemotherapy produced similar results to those achieved with allo‐HSCT in the prognosis of pediatric Ph+ ALL.^14^, ^28^, ^29^ Still, whether this strategy is applicable to adult patients with Ph+ ALL remains debatable. Clinical studies comparing allo‐HSCT and TKI combined with chemotherapy reported varied results. To clarify the issue, we collected the relevant clinical studies to conduct a meta‐analysis. The pooled result could offer the evidence for treatment decisions in adult patients with Ph+ ALL during post‐remission.

## 2 METHODS

Our meta‐analysis was conducted based on the Preferred Reporting Items for Systematic Reviews and Meta‐Analyses (PRISMA) statement.^30^

### 2.1 Study selection

In the meta‐analysis, we included all studies comparing allo‐HSCT and TKI plus chemotherapy during post‐remission after induction chemotherapy in adult Ph+ ALL patients. We omitted studies that included CML patients, studies conducted in the pre‐TKI era, and studies using pediatric patients. Other excluded studies were those with insufficient data, especially on hazard ratios (HR), and studies comprising just an abstract or brief report. In addition, minimal residual disease (MRD) status after remission was not part of the criteria for inclusion.

To identify relevant articles, we searched the Pubmed, Embase, and Web of Science databases. We also retrieved references in identified articles. We limited the date range for the studies from January 2010 to December 2020. The search strategy included the following terms: “Philadelphia chromosome‐positive acute lymphoblastic leukemia’’ OR “BCR‐ABL positive acute lymphoblastic leukemia” OR “Ph+acute lymphoblastic leukemia’’ AND “tyrosine kinase inhibitor” OR “TKI” OR “imatinib” OR “dasatinib” OR “nilotinib” OR “ponatinib” AND “hematopoietic stem cell transplantation” OR “HSCT” OR “SCT.”

### 2.2 Data collection

We extracted data consisting of author, publication year, country, sample size, TKI type, age, follow‐up duration, HR and 95% confidence interval (CI) of OS, relapse‐free survival (RFS), number of patients in non‐relapse mortality (NRM), and non‐relapsed survival (NRS). When unable to directly collect the data, we calculated the effect size based on Tierney's methods.^31^ Two independent investigators assessed the articles selected for inclusion. Any divergences in their assessments were resolved through discussion or by consulting the senior specialist. The data extraction was repeated by both investigators using the same standardized procedures. Conflicts in data extraction were also resolved through negotiation or by asking senior specialists for advice. To avoid overlap, only the most recent publication reporting the relevant outcome measures was included for each study.

### 2.3 Data statistics

We used an HR with a 95% CI to evaluate OS and RFS between allo‐HSCT and TKI plus chemotherapy. We employed the odds ratio (OR) with a 95% CI to assess NRM and NRS associated with the two treatments. *I*‐square (*I* ^2^) statistic was used to test the heterogeneity, and *I* ^2^>50% was considered significant. We performed subgroup analysis and meta‐regression when necessary. In addition, we conducted a sensitivity analysis to assess the stability of the pooled result and used Begg's test to evaluate publication bias. All analyses were conducted with Stata software 16.0 (Stata Corp, College Station, TX, USA) and Revman 5.3 (Revman the Cochrane, Collaboration, Oxford, England).

## 3 RESULT

### 3.1 Literature screening

**FIGURE 1:**
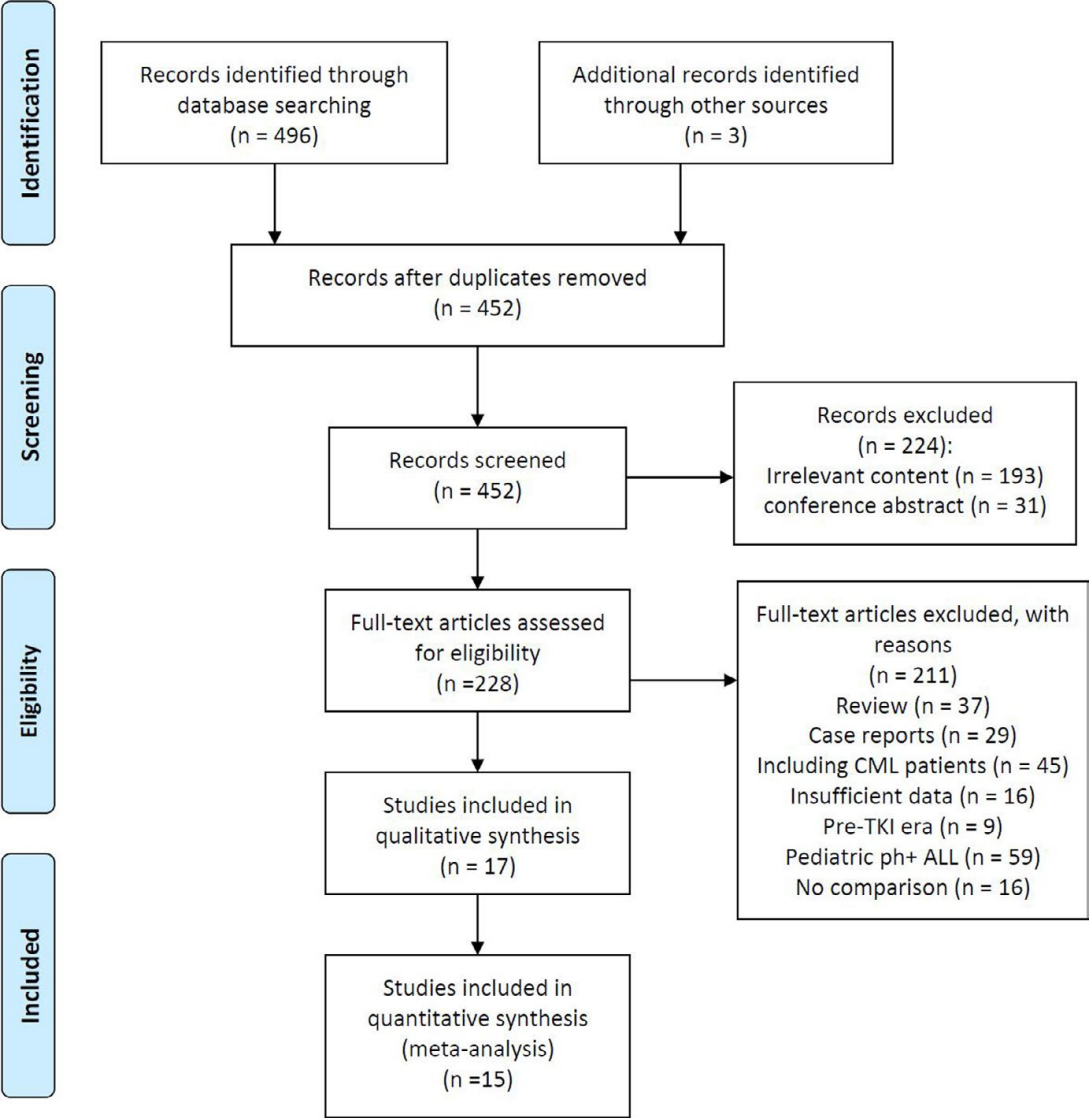
Study selection process

The flowchart of the study selection process is shown in Figure 1. A total of 499 articles were retrieved: 496 from databases and 3 from references in identified articles. Among those records, 271 articles were excluded due to being duplicated, being conference abstracts, and having irrelevant content. The remaining 228 full‐text articles were reviewed based on style, data, types of disease, and comparison. Altogether, 17 studies made it through to the next step of screening.

Next, the Newcastle‐Ottawa Scale (NOS) was used to assess the quality of the 17 studies, with a focus on data stability in retrospective studies.^32^ We defined a *quality assessment* as six points or higher for prospective studies and seven points or higher for retrospective studies. Given such criteria, two studies with low scores were excluded.^33^, ^34^ In the end, 15 qualitative publications were included in our study.

The baseline characteristics of the articles included in our meta‐analysis, all published from 2012 to 2019, are listed in Table 1. In total, the studies enrolled 959 adult Ph+ ALL patients receiving imatinib,^35^, ^36^, ^37^, ^38^, ^39^, ^40^, ^41^, ^42^, ^43^ dasatinib,^35^, ^43^, ^44^, ^45^, ^46^ nilotinib,^47^, ^48^ or ponatinib.^49^ Seven of the studies were prospective.

**TABLE 1:** Characteristics of included studies

Study	Year	Country	No. of patients (HSCT/CT)	Median age (range)	Chemotherapy Regimen in consolidation therapy	Median follow‐up (range, month)	TKI	Study type	NOS score
Agrawal	2019	India	16/30	35 (14–76)	Hyper‐CVAD	17.5 (2–58)	IM/DA	R	7
Chang	2019	China	30/40	45 (21–69)	BFM‐like	15 (1–131)	DA	R	7
Daver	2015	USA	16/23	51 (17–84)	Hyper‐CVAD	130 (73–149)	IM	P	7
Fujisawa	2017	Japan	43/22	49 (18–64)	Hyper‐CVAD	34 (13.4–52.8)	IM	P	8
Hatta	2018	Japan	59/37	45 (15–64)	HD‐Ara+MTX	58.8 (4.8–96)	IM	P	8
Jabbour	2018	USA	15/61	47 (39–61)	Hyper‐CVAD	36 (22–63,IQR)	PO	P	9
Kim	2015	Korea	57/25	47 (17–71)	DVP+HD‐Ara+VP16	NA	NL	P	9
Liu	2019	China	13/14	40 (21–59)	COATD/HD‐MTX+MA	56.5 (49–72)	NL	R	7
Ravandi	2015	USA	12/57	55 (21–80)	Hyper‐CVAD	67 (0.3–97)	DA	P	8
Ravandi	2016	USA	38/40	44 (22–60)	Hyper‐CVAD	36 (9–63)	DA	P	8
Tanguy	2013	France	24/9	45 (16–59)	VP+HD‐Ara+MTZ	29.5 (0.6–59.8)	IM	P	7
Thyagu	2012	Canada	16/12	46(18–60)	DOLD+6‐MP+MTX	85 (46–110)	IM	R	7
Togasaki	2014	Japan	13/9	53 (22–72)	Hyper‐CVAD	25 (7–126)	IM	R	7
Wang	2017	China	77/56	37 (14–65)	Hyper‐CVAD	33 (4–114)	IM	R	7
Wang	2019	China	60/74	NA (14–60)	Hyper‐CVAD	37 (7–115)	IM/DA	R	7

Abbreviations: 6‐MP, mercaptopurine; Ara, cytarabine; BFM, Berlin‐Frankfurt‐Münster; COATD, cyclophosphamide, vincristine, cytarabine, teniposide, dexamethasone; CT, chemotherapy; CVAD, cyclophosphamide, vincristine, doxorubicin, dexamethasone; DA, dasatinib; DOLD, doxorubicin, vincristine, asparaginase, dexamethasone; DVP, daunorubicin, vincristine, prednisone; HSCT, hematopoietic stem cell transplantation; IM, imatinib; IQR, interquartile range; MA, methotrexate, cytarabine; MTX, methotrexate; MTZ, mitoxantrone; NA, not available; NL, nilotinib; NOS, Newcastle‐Ottawa Scale; P, prospective, PO, ponatinib; R, retrospective; TKI, tyrosine kinase inhibitors; VP16, etoposide.

### 3.2 Survival analysis of OS and RFS

**FIGURE 2:**
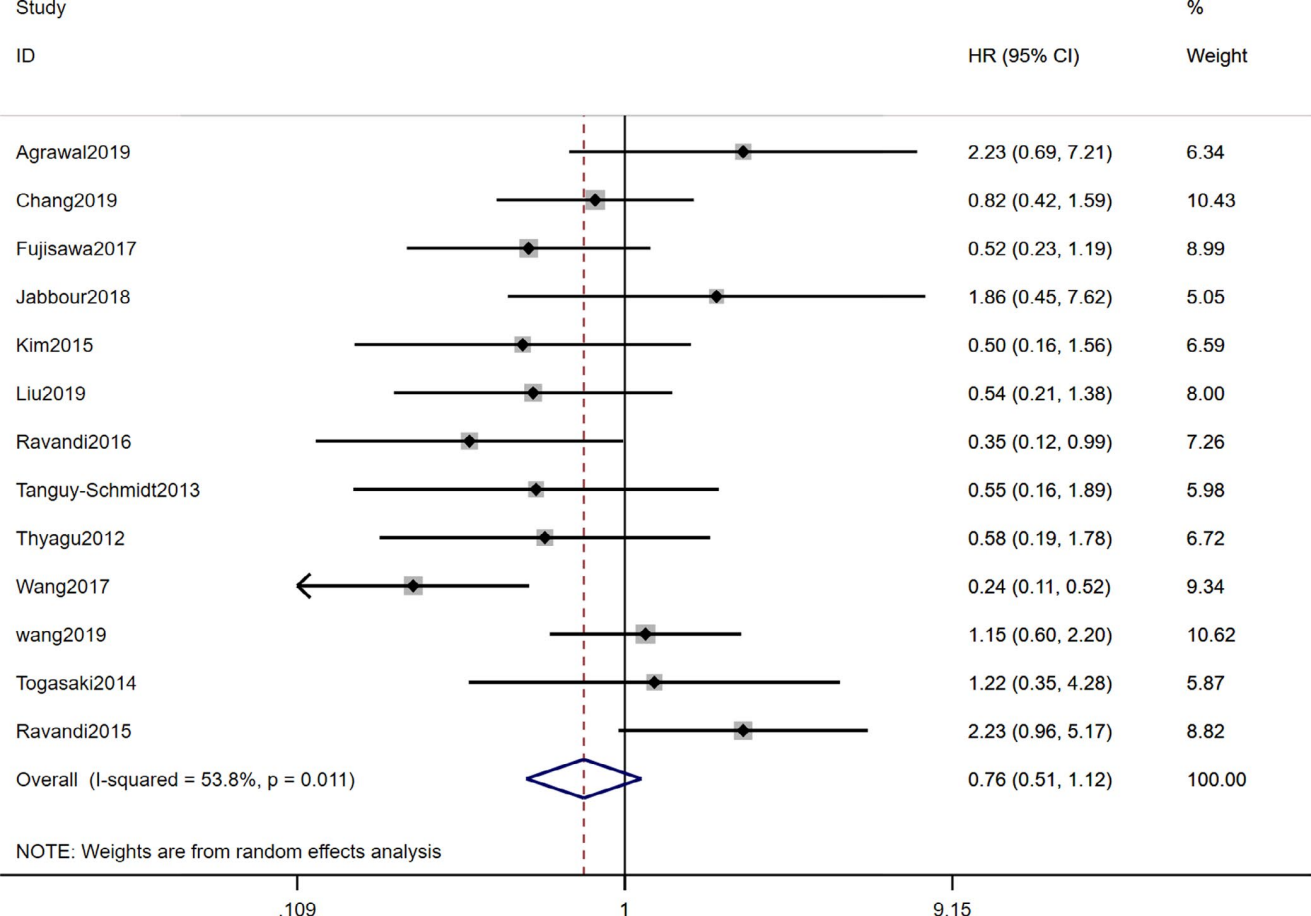
Forest plots of studies evaluating OS of comparison between Allo‐HSCT and TKI+chemotherapy in adult patients with Ph+ ALL

**FIGURE 3:**
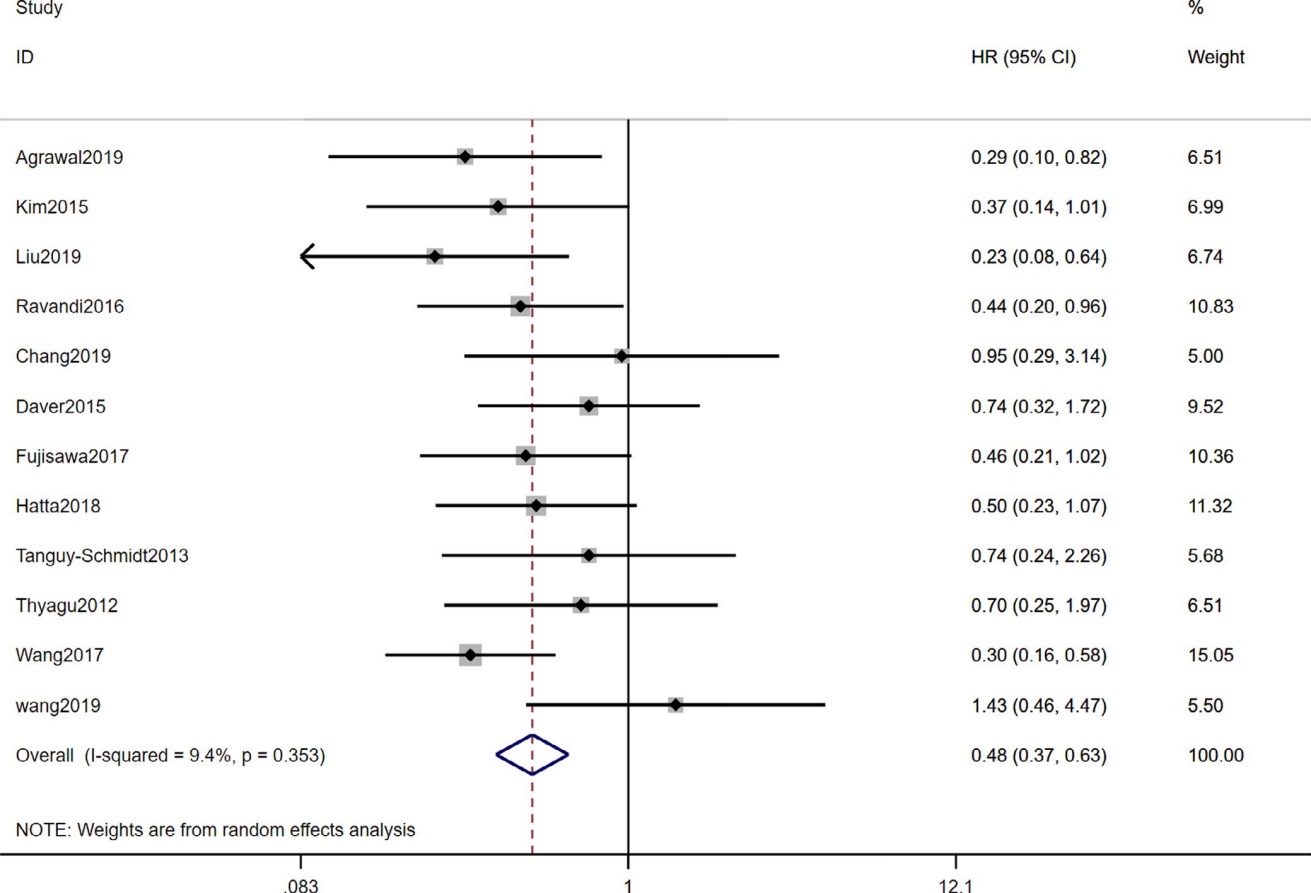
Forest plots of studies evaluating RFS of comparison between Allo‐HSCT and TKI+chemotherapy in adult patients with Ph+ ALL

All included studies reported the Kaplan–Meier (K–M) survival curve or an HR with a 95% CI for OS or RFS. In terms of OS, two studies^36^, ^38^ were excluded due to no HR being reported for the TKI cohort, and the pooled result of the remaining 13 studies showed that there was no difference between TKI combined with chemotherapy and allo‐HSCT in post‐remission (HR=0.76, 95% CI [0.51–1.12], *p*=0.16). However, a significant heterogeneity with I^2^=53.8% existed among the studies (*p*=0.01; Figure 2). To examine RFS, we incorporated the HRs and 95% CIs of another 12 studies (three excluded studies^22^, ^41^, ^45^ did not report the outcome data of patients in RFS). The pooled result showed that, statistically, patients undergoing allo‐HSCT had a longer RFS than those receiving TKI plus chemotherapy (HR = 0.48, 95% CI [0.37–0.63], *p *= 0.00) without significant heterogeneity (*I* ^2^ = 9.4%, *p *= 0.35; Figure 3).

### 3.3 Odds ratio of NRM and NRS

**FIGURE 4:**
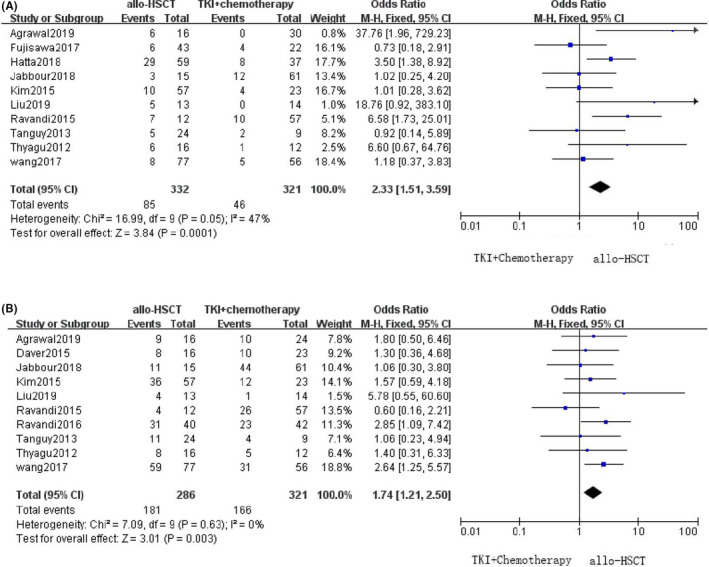
Forest plots for the comparison of Allo‐HSCT With TKI+chemotherapy in NRM and NRS. (A. NRM; B. NRS)

In addition to OS and RFS, NRM, and NRS are important indices in cancer research. A total of 10 studies reported the NRM numbers in both groups. In the allo‐HSCT group (332 patients), 85 patient deaths were not the result of the disease itself while in the TKI plus chemotherapy group (321 patients), 46 patient deaths were not attributed to the disease. We calculated OR and 95% CI and found that NRM occurred significantly more frequently in patients undergoing allo‐HSCT than in patients receiving TKI plus chemotherapy (OR = 2.33, 95% CI [1.51–3.59], *p *= 0.00). Another 10 studies reported the number of patients with NRS: in the allo‐HSCT group, 181 out of 286 patients survived, and in the TKI plus chemotherapy group, 166 out of 321 patients survived. In contrast, as seen in Figure 4, significantly more patients survived without recurrence after allo‐HSCT therapy (OR = 2.64, 95% CI [1.25–5.57], *p *= 0.00).

### 3.4 Subgroup analysis and meta‐regression

As a result of notable heterogeneity in OS, we continued to conduct subgroup analysis and meta‐regression to identify the root of the heterogeneity. The subgroup analysis was performed based on age, donor type, TKI type, study design, chemotherapy regimen, location, sample size, and median follow‐up period. As shown in results presented in Table 2, the heterogeneity did not stem from these variables. The meta‐regression also showed that the heterogeneity did not originate from age (*p *= 0.45), donor type (*p *= 0.96), TKI type (*p *= 0.32), study design (*p *= 0.82), chemotherapy regimen (*p* = 0.41), location (*p* = 0.46), sample size (*p* = 0.06), or median follow‐up period (*p* = 0.51).

**TABLE 2:** Subgroup analysis for OS

Subgroup	No. of study	No. of patients (HSCT/CT)	HR (95% CI)	*p* value	Heterogeneity
Age					
Range≤60y	5	151/149	0.68 (0.43–1.06)	0.086	*I* ^2^ = 12.3%; *p* = 0.336
Range >60y	8	263/300	0.87 (0.47–1.62)	0.660	*I* ^2^ = 72.6%; *p *= 0.001
Donor type					
HLA‐matched	6	146/149	0.75 (0.41–1.36)	0.347	*I* ^2^ = 50.4%; *p *= 0.073
HLA‐matched /HLA‐mismatched	7	268/300	0.76 (0.42–1.38)	0.365	*I* ^2^ = 69.7%; *p* = 0.003
Location					
East	8	309/270	0.69 (0.42–1.14)	0.150	*I* ^2^ = 63.1%; *p* = 0.008
West	5	105/179	0.86 (0.40–1.87)	0.711	*I* ^2^ = 58.8%; *p* = 0.046
Sample size					
HSCT>CT	6	230/133	0.46 (0.29–0.74)	0.001	*I* ^2^ = 25.6%; *p* = 0.242
HSCT<CT	7	184/316	1.04 (0.64–1.68)	0.086	*I* ^2^ = 49.8%; *p* = 0.063
TKI agents					
Imatinib	5	156/126	0.48 (0.27–0.83)	0.008	*I* ^2^ = 39.8%; *p* = 0.156
Dasatinib	3	80/154	0.89 (0.35–2.30)	0.811	*I* ^2^ = 73.5%; *p* = 0.023
Nilotinib	2	70/39	0.52 (0.25–1.08)	0.080	*I* ^2^ = 0%; *p* = 0.926
Ponatinib	1	15/61	1.86 (0.45–7.60)	0.388	/
Study design					
Prospective	6	225/235	0.76 (0.40–1.46)	0.414	*I* ^2^ = 56.1%; *p* = 0.044
Retrospective	7	189/214	0.74 (0.42–1.31)	0.307	*I* ^2^=67.5%; *p* = 0.005
Chemotherapy regimen					
Hyper‐CVAD A/B	8	270/349	0.88 (0.46–1.69)	0.696	*I* ^2^ = 75.7%; *p* = 0.000
Others	5	140/100	0.64 (0.42–0.98)	0.038	*I* ^2^ = 0%; *p* = 0.922
Median follow‐up					
≥36 months	6	171/240	0.91 (0.51–1.61)	0.741	*I* ^2^ = 53.2%; *p* = 0.058
<36 months	6	186/184	0.67 (0.35–1.27)	0.218	*I* ^2^ = 66.7%; *p* = 0.010

Abbreviations: CI, confidential interval; CT, chemotherapy; CVAD, cyclophosphamide, vincristine, doxorubicin, dexamethasone; HLA, human leukocyte antigen; HR, hazard ratio; HSCT, hematopoietic stem cell transplantation; OS, overall survival; TKI, tyrosine kinase inhibitors.

### 3.5 Sensitivity analysis

**FIGURE 5:**
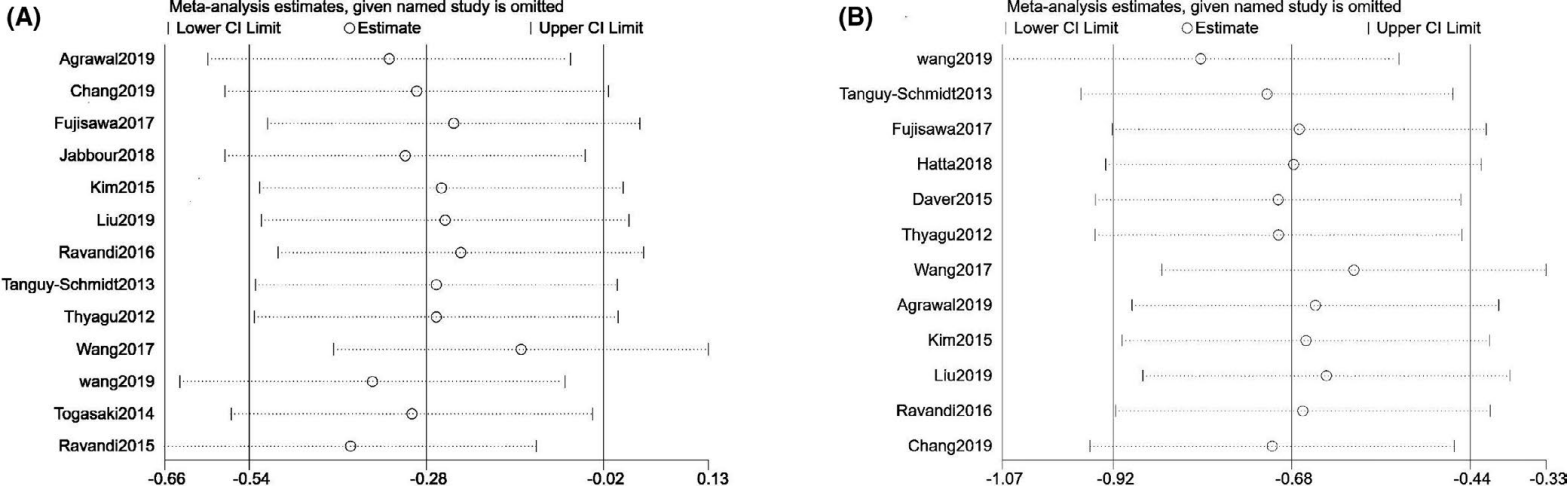
Sensitivity analysis on the comparison between Allo‐HSCT and TKI +chemotherapy in adult patients with Ph+ ALL. (A. OS; B. RFS)

To evaluate the stability of the pooled results, we performed sensitivity analysis. The result demonstrated that our pooled results would remain steady even if any included study was omitted in both RFS and OS (Figure 5).

### 3.6 Publication bias

**FIGURE 6:**
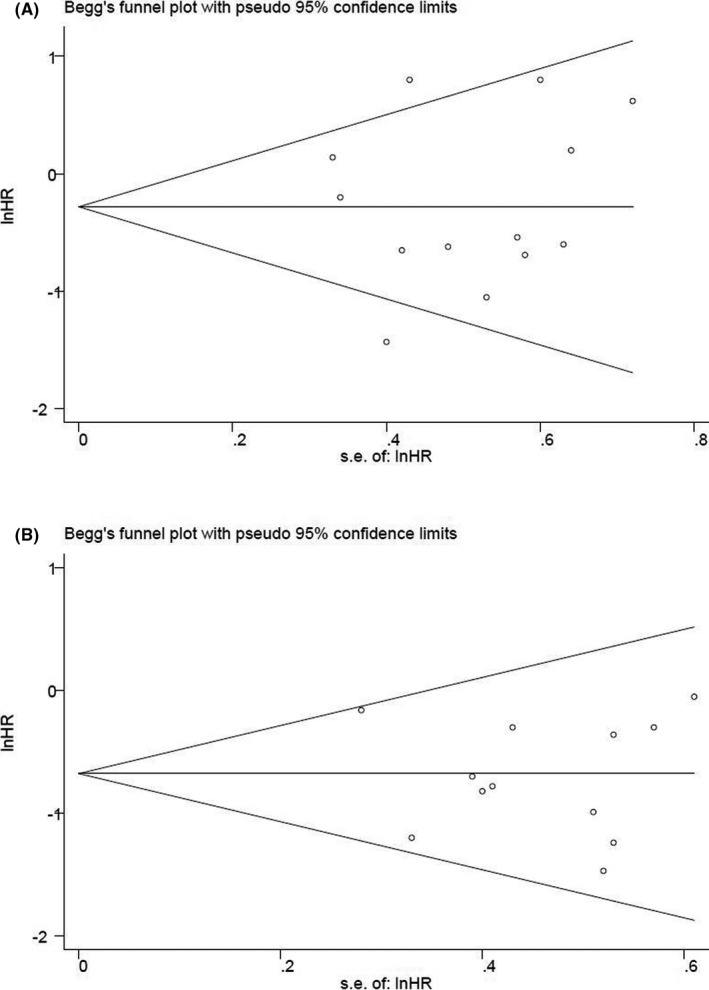
Funnel plots for publication bias (A. OS outcome for patients receiving TKI +chemotherapy versus undergoing Allo‐HSCT; B. RFS outcome for patients receiving TKI +chemotherapy versus undergoing Allo‐HSCT)

We adopted Begg's test to detect publication bias. Among the included studies, no publication bias was found in OS (*p* = 0.43) and RFS (*p* = 0.63) (Figure 6).

## 4 DISCUSSION

We analyzed 15 studies, which collectively included 473 patients undergoing allo‐HSCT and 486 patients receiving TKI combined with chemotherapy in post‐remission. The results showed that both treatments have statistically and clinically advantages. TKI combined with chemotherapy and allo‐HSCT showed no difference in effect on OS. In terms of RFS, TKI combined with chemotherapy did worse than allo‐HSCT. NRM occurred more frequently in the allo‐HSCT patients than in those undergoing TKI plus chemotherapy. The NRS rates in patients in post‐transplantation were higher than those in post‐remission therapy with TKI plus chemotherapy. These results suggest that, in the TKI era, allo‐HSCT is no longer necessary: TKI combined with chemotherapy results in similar long‐term survival rates for adult patients with Ph+ ALL in post‐remission. Our findings may help to determine the most appropriate treatment plans for patients achieving HCR after induction chemotherapy.

Existing literature reviews have increasingly prompted questions about the choice of allo‐HSCT as the preferred treatment for adult Ph+ ALL patients after induction chemotherapy.^2^, ^50^, ^51^, ^52^, ^53^, ^54^ TKI combined with chemotherapy as treatment in post‐remission has advance to align with the efficacy of allo‐HSCT in adult patients. The literature reviews either proposed that TKI plus chemotherapy in treatment after remission for adult patients was an option that should be considered or suggested that the necessity of allo‐HSCT in the treatment of Ph+ ALL has decreased in the TKI era. However, all the reviews were based on past studies and reports of clinical experience. Currently, there is no evidence‐based medicine (EBM) supporting the view in the literature.

A meta‐analysis concerning the comparison of allo‐HSCT and TKI combined with chemotherapy in adult Ph+ ALL patients was published recently.^55^ The results showed that the OS and disease‐free survival (DFS) of patients receiving TKI plus chemotherapy as a treatment after remission were both shorter than those of patients undergoing allo‐HSCT. In the study, OR was employed as effect size, and the time factor was no considered in the merging process. Significant heterogeneity also existed in the pooled DFS (I^2^ = 62%) and OS (*I* ^2^ = 59%) rates. Our meta‐analysis obviously differed from the recent meta‐analysis. In our study, the patient survival time was considered, and HR was set to the effect size. Therefore, we abandoned some articles from which HR could not be obtained. Our results showed no difference in OS between patients receiving TKI plus chemotherapy and those undergoing allo‐HSCT; in terms of RFS, our results mirrored those in the published article. We also incorporated the NRM and NRS of all patients. The findings showed that the elevated NRM in the allo‐HSCT group resulted in a similar OS among those in the TKI plus chemotherapy group, a finding consistent with many published articles.^8^, ^56^ In addition to the toxicity of chemotherapy drugs, patients also encountered complications after allo‐HSCT, including engrafting failure, GvHD, infection, and relapse, any of which can be fatal. Thus, in terms of OS, allo‐HSCT does not appear to benefit adult Ph+ ALL patients more than TKI combined with chemotherapy. For the reasons above, our study results seemed closer to the real world compared to the published meta‐analysis.

In our study, heterogeneity in OS was an obvious flaw (*I* ^2^ = 53.8%), though the root of the heterogeneity was not identified through meta‐regression and subgroup analysis. We contemplated whether the factors that cannot be separated from the articles into meta‐regression and subgroup analysis, such as age and complete molecular remission (CMR) status, caused the heterogeneity. Although we also concluded no different OS was found between TKI plus chemotherapy and allo‐HSCT in patients younger than 60 years from a subgroup analysis, a difference in average patient age in the two treatment groups has always been problematic because in the real world, elderly patients almost always receive TKI plus chemotherapy as a treatment in post‐remission and research on the use of allo‐HSCT versus TKI plus chemotherapy in elderly Ph+ ALL patients is lacking,^57^ A meta‐analysis that included 2,962 patients with Ph‐ ALL previously showed that only patients younger than 35 years old benefited from allo‐HSCT.^58^ On the other hand, most articles comparing treatments in post‐remission for Ph+ ALL patients have had the same premise: the treatment goal of achieving CMR. These studies concluded that the prognosis in terms of CMR is similar for patients treated with TKI combined with chemotherapy as a treatment in post‐remission and for those undergoing allo‐HSCT.^8^, ^14^, ^20^, ^59^, ^60^ However, among the studies included in our meta‐analysis, only Wang's (2019) study^43^ reported the molecular remission status of patients after remission; the other studies did not provide such data in their articles. Without considering CMR status, our pooled result showed no significant difference in OS between allo‐HSCT and TKI plus chemotherapy groups. Yet Wang's (2019) study^43^ confirmed that, regardless of whether patients achieve CMR after remission, OS was similar between the two treatment groups. Notably, when patients without CMR were excluded, the K‐M survival curves of the two treatment groups were almost equivalent. Molecular remission status may have caused some heterogeneity in the process of merging results.

Additionally, there are other limitations in interpreting our findings. First, the sample size was small, with our meta‐analysis including fewer than 1,000 patients. A small sample size may result in bias. In the future, we could update our meta‐analysis by extending the publishing date. Second, retrospective studies were included in our meta‐analysis. Compared with prospective studies, retrospective studies are insufficiently objective and lack uniform standards. Therefore, we set strict inclusion criteria for retrospective studies. Subgroup analysis and meta‐regression have also shown no indication that study‐design type contributes to the heterogeneity. Third, in addition to allo‐HSCT and TKI plus chemotherapy, other post‐remission treatments—such as autologous‐HSCT,^61^, ^62^ blinatumomab,^63^, ^64^, ^65^ inotuzumab ozogamicin,^66^, ^67^ and chimeric antigen receptor t‐cell immunotherapy (CAR‐T)^68^, ^69^, ^70^—are available. We also searched for studies on these treatments. A few studies looked at the limited number of patients undergoing autologous‐HSCT. Two studies comparing allo‐HSCT to auto‐HSCT in Ph+ ALL patients showed no difference in RFS and OS between the two groups.^61^, ^62^ Blinatumomab, inotuzumab ozogamicin, and CAR‐T, as immunotherapy, are usually employed in relapsed or refractory patients and are not used in routine post‐remission treatment. Fourth, the year of transplantation and graft source were not evaluated due to the lack of data in the articles. The analysis and discussion should be conducted in further studies at a future date. Fifth, all included studies did not take quality of life into consideration. Although we concluded no different OS between TKI plus chemotherapy and allo‐HSCT for adult Ph+ ALL, quality of life was not discussed. In the future, quality of life can be analyzed by special scales during follow‐up.

## 5 CONCLUSION

Current studies concluded that to a certain extent, TKI combined with chemotherapy can provide adult Ph+ ALL patients with a similar OS rate as allo‐HSCT in the TKI era. The results of our meta‐analysis also indicate that patients receiving TKI combined with chemotherapy in post‐remission had a shorter RFS than patients receiving allo‐HSCT. Our results provide EBM support to the assertion that TKI plus chemotherapy in post‐remission leads to an outcome no worse than allo‐HSCT in adult patients with Ph+ ALL. In summary, TKI combined with chemotherapy can be considered for adult Ph+ ALL patients ineligible for allo‐HSCT in post‐remission. To validate this finding, more prospective studies with large sample sizes and the inclusion of CMR status should be carried out in the future.

## CONFLICT OF INTEREST

The authors declare no conflict of interest.

## Data Availability

The data and materials analyzed in our study are available from the corresponding author upon reasonable request.
